# Quantum walk hydrodynamics

**DOI:** 10.1038/s41598-019-40059-x

**Published:** 2019-02-27

**Authors:** Mohamed Hatifi, Giuseppe Di Molfetta, Fabrice Debbasch, Marc Brachet

**Affiliations:** 1 Aix-Marseille Université, CNRS, École Centrale de Marseille, Institut Fresnel UMR 7249, 13013 Marseille, France; 2 Aix-Marseille Université, Université de Toulon, CNRS, LIS, France, Natural Computation research group, Marseille, France; 3 Departamento de Física Teórica and IFIC, Universidad de Valencia-CSIC, Dr. Moliner 50, 46100 Burjassot, Spain; 4 LERMA, UMR 8112, UPMC and Observatoire de Paris, 61 Avenue de l’Observatoire, 75014 Paris, France; 5 Laboratoire de Physique Statistique, École Normale Supérieure, PSL Research University, UPMC Univ Paris 06, Sorbonne Universités, Université Paris Diderot, Sorbonne Paris-Cité, CNRS, 24 Rue Lhomond, 75005 Paris, France

## Abstract

A simple Discrete-Time Quantum Walk (DTQW) on the line is revisited and given an hydrodynamic interpretation through a novel relativistic generalization of the Madelung transform. Numerical results show that suitable initial conditions indeed produce hydrodynamical shocks and that the coherence achieved in current experiments is robust enough to simulate quantum hydrodynamical phenomena through DTQWs. An analytical computation of the asymptotic quantum shock structure is presented. The non-relativistic limit is explored in the Supplementary Material (SM).

## Introduction

Quantum walks (DTQWs) are unitary quantum automata that can be viewed as formal generalizations of classical random walks. Following the seminal work of Feynman^1^ and Aharonov^2^ they were considered in a systematic way by Meyer^3^. DTQWs have been realized experimentally with a wide range of physical objects and setups^4–10^, and are studied in a large variety of contexts, ranging from quantum optics^10^ to quantum algorithmics^11,12^, solid-state physics^13–16^ and biophysics^17,18^. The aim of this Letter is to show on a simple example, through both literal and numerical computations, that QW dynamics can be mapped unto quantum fluid dynamics (QFD) and that QWs can thus be used to model and experimentally simulate quantum fluids. In particular, DTQWs are thus natural candidates for future laboratory simulation of matter wave interferences in quantum fluids, dispersive hydrodynamics^19^ and extreme astrophysical plasmas (see e.g. §3.2.3 and §3.2.4 in^20^).

We focus on a simple spatially homogeneous and time independent DTQW on the line whose continuous limit is identical to the free Dirac equation in flat 2D space-time. We then introduce a new relativistic generalization of the Madelung transform which maps this Dirac equation into a 2D dispersive hydrodynamics for relativistic quantum fluids. In the non relativistic limit, the two component spinor which obeys the Dirac equations degenerates into a single wave-function which obeys the Schrödinger equation, which can also be viewed as the continuous space limit of a continuous time quantum walk. The relativistic Madelung transform then becomes the usual Galilean Madelung transform. To prove that the hydrodynamical vision goes beyond a mere rewriting of the equations, we demonstrate through direct numerical simulations that the DTQW can actually model QFD shocks. We also present an analytical computation of the asymptotic Galilean shock structure through Pearcey integral commonly used in Optics.

## Methods

### The DTQW

The Hilbert space of the DTQW is the tensor product ${ {\mathcal H} }_{p}\otimes { {\mathcal H} }_{s}$, where ${ {\mathcal H} }_{p}$ is the discrete line with basis *n*, $n\in {\mathbb{Z}}$ and ${ {\mathcal H} }_{s}$ is the ‘spin’-space with basis vectors $|L\rangle ={(1,0)}^{T}$ and $|R\rangle ={(0,1)}^{T}$. The evolution is controlled by the unitary operator $U=TC$, where $T={\sum }_{n}\,[|n-1,L\rangle \,\langle n,L|+|n+1,R\rangle \,\langle n,R|]$ is the translation operator and $C={e}^{-i\theta {\sigma }_{1}}$ is the quantum coin operator defined from the first Pauli matrix *σ*_1_ and an arbitrary constant angle *θ*. The explicit evolution equation of the walk reads:

1 $$[\begin{array}{c}{{\rm{\Psi }}}_{L}(l+1,n-1)\\ {{\rm{\Psi }}}_{R}(l+1,n+1)\end{array}]=[\begin{array}{cc}\cos \,\theta  & -i\,\sin \,\theta \\ -i\,\sin \,\theta  & \cos \,\theta \end{array}]\,[\begin{array}{c}{{\rm{\Psi }}}_{L}(l,n)\\ {{\rm{\Psi }}}_{R}(l,n)\end{array}]$$

where the index *l* represents the iteration or discrete time.

### Continuous Limit

Introduce now two positive real numbers *m* and $\varepsilon$, choose $\theta (\varepsilon ,m)=\varepsilon m$, consider that ${{\rm{\Psi }}}_{L/R}(l,n)$ are the values taken by some differentiable functions ${{\rm{\Psi }}}_{L/R}(t,x)$ at point ${t}_{l}=l\varepsilon$ and ${x}_{n}=n\varepsilon$. Equation (1) then admits a continuous limit which coincides with the Dirac equation^21^

2 $$i{\gamma }^{\mu }{\partial }_{\mu }\psi -m\psi =0,$$

where $\psi ={({{\rm{\Psi }}}_{L},{{\rm{\Psi }}}_{R})}^{T}$, ${\gamma }^{0}={\sigma }_{1}$, ${\gamma }^{1}=i{\sigma }_{2}$ (*σ*_2_ is the second Pauli matrix) and $\hslash =c=1$. The mass *m* is thus homogeneous to the inverse of a length.

Dirac Eq. (2) can be obtained from the Lagrangian density $$ {\mathcal L} =\frac{i}{2}(\overline{\psi }{\gamma }^{\mu }{\partial }_{\mu }\psi -{\partial }_{\mu }\overline{\psi }{\gamma }^{\mu }\psi )-m\overline{\psi }\psi $$ where $$\overline{\psi }={\psi }^{\dagger }{\gamma }^{0}$$. The associated particle current is $${j}^{\mu }=\overline{\psi }{\gamma }^{\mu }\psi $$ and the stress energy tensor reads $${T}^{\mu \nu }=\frac{i}{4}[\overline{\psi }{\gamma }^{\mu }{\partial }^{\nu }\psi -$$$${\partial }^{\nu }\overline{\psi }{\gamma }^{\mu }\psi +(\mu \leftrightarrow \nu )]$$. Both *j* and *T* are conserved *i*.*e*. $${\partial }_{\mu }{T}^{\mu \nu }=0$$ and $${\partial }_{\mu }{j}^{\mu }=0$$. Note that the above Lagrangian density leads to a symmetric canonical stress-energy tensor.

## Results

### New variables

The definition of *j* leads to $${j}^{0}=|{\psi }_{R}{|}^{2}+|{\psi }_{L}{|}^{2}$$ and $${j}^{1}=|{\psi }_{R}{|}^{2}-|{\psi }_{L}{|}^{2}$$. Note that $${({j}^{0})}^{2}-{({j}^{1})}^{2}=$$$$4|{\psi }_{L}{|}^{2}|{\psi }_{R}{|}^{2}\ge 0$$ so that the current *j* is necessarily timelike or null. We then introduce $${\phi }_{\pm }={\phi }_{L}\pm {\phi }_{R}$$ where $${\phi }_{L/R}$$ is the phase of $${{\rm{\Psi }}}_{L/R}$$ and replace the variables $$(|{\psi }_{L}{|}^{2},|{\psi }_{R}{|}^{2},{\varphi }_{L},{\varphi }_{R})$$ by $$({j}^{0},{j}^{1},{\phi }_{+},{\phi }_{-})$$. In particular, the spinor $$\psi $$ now reads

3 $$\psi ({\bf{x}},t)=\frac{1}{\sqrt{2}}{e}^{i{\phi }_{+}/2}[\begin{array}{c}\sqrt{{j}^{0}-{j}^{1}}{e}^{i{\phi }_{-}/2}\\ \sqrt{{j}^{0}+{j}^{1}}{e}^{-i{\phi }_{-}/2}\end{array}]$$

and ${\phi }_{+}/2$ can be viewed as the global phase of $\psi$.

In terms if these new variables, the Lagrangian density and the stress energy tensor read $$ {\mathcal L} =-\,m{({j}_{\mu }{j}^{\mu })}^{1/2}$$$$\cos \,{\phi }_{-}-\frac{1}{2}({j}^{\mu }{\partial }_{\mu }{\phi }_{+}-{\varepsilon }^{\mu \nu }{j}_{\nu }{\partial }_{\mu }{\phi }_{-})$$ and $${T}^{\mu \nu }=-\,\frac{1}{4}({j}^{\mu }{\partial }^{\nu }{\phi }_{+}-{\varepsilon }^{\mu \alpha }{j}_{\alpha }{\partial }^{\nu }{\phi }_{-}+(\mu \leftrightarrow \nu ))$$, where $${\varepsilon }^{\mu \nu }$$ denotes the completely antisymmetric symbol of rank two, with the convention $${\varepsilon }^{01}=-\,{\varepsilon }^{10}=1$$.

The dynamical equations derived from ${\mathcal L} ({j}^{0},{j}^{1},{\phi }_{+},{\phi }_{-})$ are

4 $${{\varepsilon }^{\mu }}_{\alpha }{\partial }_{\mu }\,{j}^{\alpha }=2m{({j}_{\mu }{j}^{\mu })}^{1/2}\,\sin \,{\phi }_{-}$$

5 $$m\,\cos \,{\phi }_{-}\,{j}^{\mu }=-\,\frac{1}{2}\,{({j}_{\mu }{j}^{\mu })}^{1/2}\,({\partial }^{\mu }{\phi }_{+}+{\varepsilon }^{\mu \nu }{\partial }_{\nu }{\phi }_{-})$$

6 $${\partial }_{\mu }{j}^{\mu }=0.$$

### Dirac quantum hydrodynamics

Since *j* is time-like or null, one can define the density *n* of the (1 + 1)D Dirac fluid by $n={({j}_{\mu }{j}^{\mu })}^{1/2}$. We now suppose that *j* is not null and define the vector $u=j/n$ as the 2-velocity of the fluid, normed to unity. The two variables *j*^0^ and *j*^1^ can then be replaced by *n* and *u*^1^ *i*.*e*. the density and the spatial part of the fluid 2-velocity. Equation (5) can then be re-written as $m\,\cos \,{\phi }_{-}\,{u}^{\mu }=-\,\frac{1}{2}\,({\partial }^{\mu }{\phi }_{+}+{\varepsilon }^{\mu \nu }{\partial }_{\nu }{\phi }_{-})$ and, in this form, brings to mind the standard relation $\frac{w}{n}{u}^{\mu }=-\,{\partial }^{\mu }\phi$ which links the velocity *u* of a relativistic potential flow to its potential $\phi$, the enthalpy per unit volume *w* and the particle density *n*. We thus retain $w=mn\,\cos \,{\phi }_{-}$ as the enthalpy per unit volume of the (1 + 1)D Dirac fluid. The velocity field *u* then derives from two potentials. One is ${\phi }_{+}/2$ *i*.*e*. the global phase of the spinor $\psi$ and contributes to *u* in the standard way. The other potential is the phase differential ${\phi }_{-}/2$ and contributes to *u* in a non-standard way, by contraction of its gradient with the (1 + 1)D completely antisymmetric symbol.

Using ((5)), one then finds that

7 $${T}^{\mu \nu }=w{u}^{\mu }{u}^{\nu }+\frac{n}{2}({\varepsilon }^{\mu \alpha }{u}_{\alpha }{\partial }^{\nu }{\phi }_{-}+{u}^{\mu }{\varepsilon }^{\nu \alpha }{\partial }_{\alpha }{\phi }_{-}),$$

to be compared with the stress-energy tensor ${T}^{\mu \nu }=w{u}^{\mu }{u}^{\nu }-p{\eta }^{\mu \nu }$ of a relativistic perfect fluid of pressure *p*. The pressure of the Dirac fluid thus vanishes. This is not surprising because classical pressure in spin-0 superfluids is generated by non-linearities^22–24^ in the wave equation and the free Dirac Eq. (2) is linear.

The last two terms on the right-hand side of (7) depend on the gradient of ${\phi }_{-}$ and, thus, on the gradient of *w*/*n*. Indeed, the definition of *w* leads to ${\sin }^{2}\,{\phi }_{-}=1-{(\frac{w}{mn})}^{2}$ and $\sin \,{\phi }_{-}d{\phi }_{-}=-\,d(\frac{w}{mn})$, so that, if $w\ne nm$,

8 $${\partial }_{\mu }{\phi }_{-}=-\,\sigma \frac{{\partial }_{\mu }(\frac{w}{mn})}{{(1-{(\frac{w}{mn})}^{2})}^{1/2}}$$

where *σ* is the sign of $\sin \,{\varphi }_{-}$. As for relativistic spin 0 superfluids^25^, the two extra-terms in the above expression of the stress-energy tensor thus depend on the gradient of a thermodynamic function (the enthalpy per particle *w*/*n*) and are therefore best viewed as generalized ‘quantum pressure’ terms. As shown in the SM the two component spinor which obeys Dirac equation degenerates, in the Galilean limit, into a single wave-function which obeys the Schrödinger equation and the relativistic hydrodynamics degenerates into the standard^26–28^ Madelung hydrodynamics.

### Numerical shock simulation

The above generalization of the Madelung transform strongly suggests that the original DTQW can be used to simulate quantum flows. First note that a general positive energy plane wave solution of (2) can be written as (see (3–6)) ${j}^{0}=n\sqrt{1+{q}^{2}}$, ${j}^{1}=nq$, ${\phi }_{+}/2=-\,m(\sqrt{1+{q}^{2}}t-qx)$, ${\phi }_{-}=0$, where *q* denotes both wave-number and momentum in unit of *m* (remember $\hslash =c=1$). The spinor

9 $${{\rm{\Psi }}}_{L}=\sqrt{\sqrt{1+{q}^{2}}-q}{e}^{im\varphi }/\sqrt{2}\,{{\rm{\Psi }}}_{R}=\sqrt{\sqrt{1+{q}^{2}}+q}{e}^{im\varphi }/\sqrt{2}$$

thus describes, at $t=0$, a unit density fluid ($n=1$) in motion with constant velocity *u*^1^ given by ${u}^{1}=q=\partial \varphi /\partial x$.

**Figure 1:**
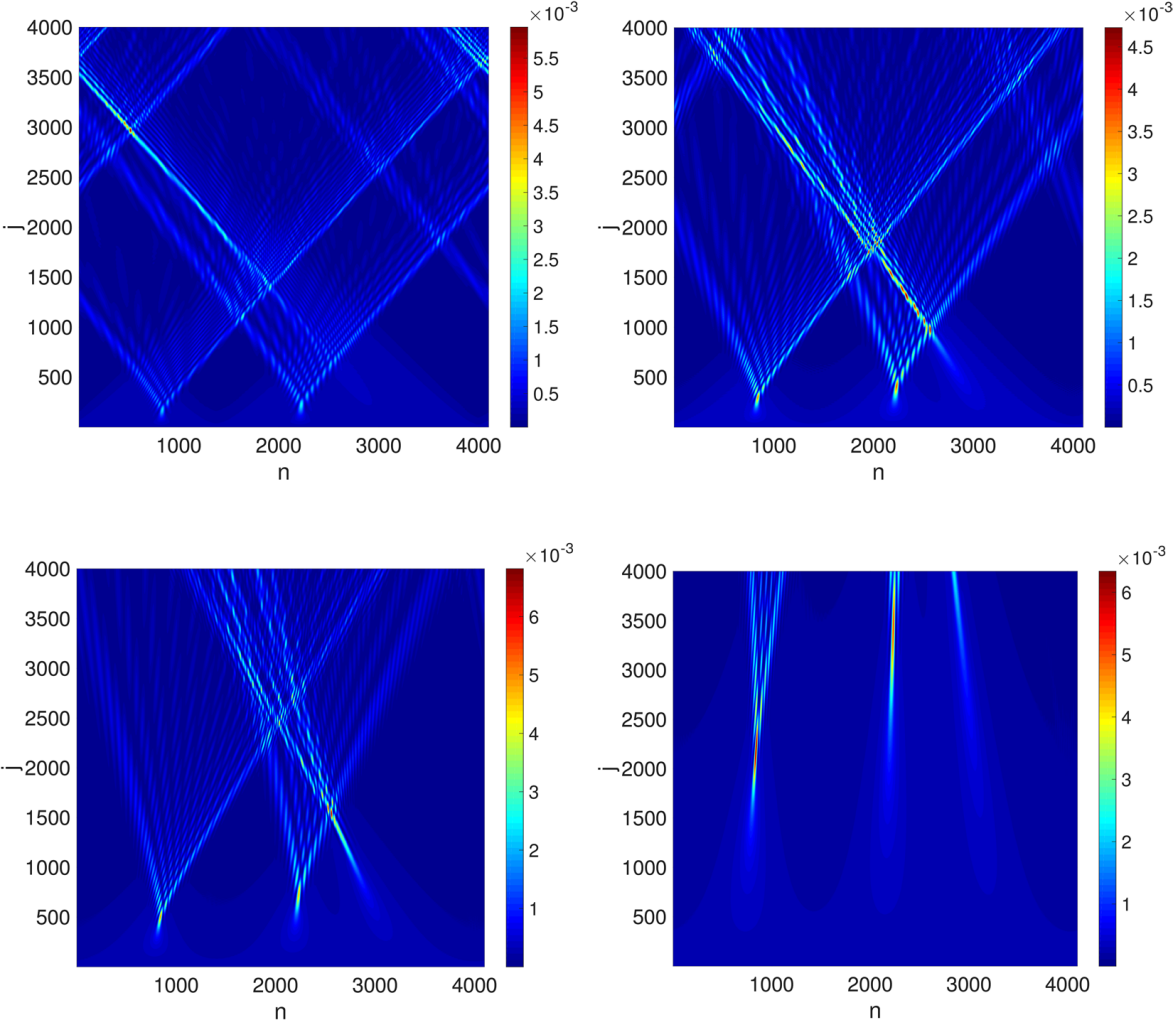
Unitary evolution of density ${j}^{0}=|{{\rm{\Psi }}}_{L}{|}^{2}+|{{\rm{\Psi }}}_{R}{|}^{2}$ for the DTQW defined in Eq. (1) with initial conditions: $\varphi ={q}_{{\rm{\max }}}[\cos (x)+\frac{1}{3}\,\cos (3x)+\frac{1}{2}\,\cos (2x+0.9)]/m$ where $m\,:\,=25.6$, 64, 128 and 512, ${q}_{{\rm{\max }}}=51.2$ and ${u}_{{\rm{\max }}}=2$, 0.8, 0.4 and 0.1 (see Eqs (9) and (10)). The grid has $N={2}^{12}$ points, $\varepsilon =2\pi /N$.

**Figure 2:**
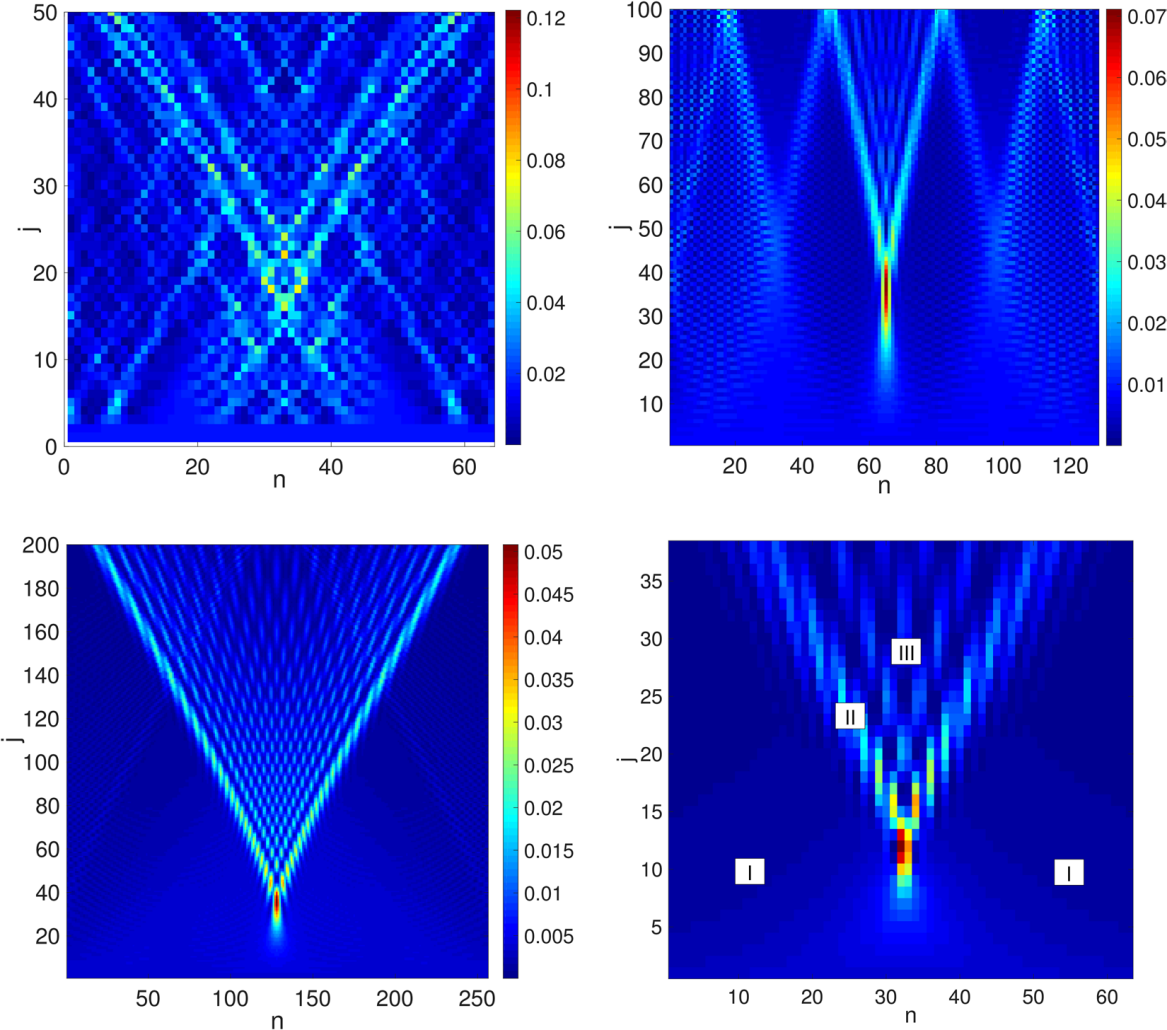
(**a**–**c**) DTQWs with initial conditions $\varphi \sim \,\cos (x)$, ${u}_{{\rm{\max }}}=0.2$ and number of grid points $n=64$ (**a**), $n=128$ (**b**), $n=256$ (**c**,**d**) Approximation by Pearcey’s integral in (*x*, *t*) space with $\varepsilon =0.05$ and zones of validity of approximations (see text).

In order to simulate quantum flows, we now select the initial conditions of the form (9) but with

10 $$\varphi =\frac{{q}_{{\rm{\max }}}}{m}[\cos (x)+\frac{1}{3}\,\cos (3x)+\frac{1}{2}\,\cos (2x+0.9)],$$

with ${q}_{{\rm{\max }}}=m{u}_{{\rm{\max }}}$ this choice corresponds to the velocity field ${u}^{1}={u}_{{\rm{\max }}}(\sin (x)-\,\sin (3x)-\,\sin (2x+0.9))$. These initial conditions are inspired by the similar (but somewhat simpler) choice $\varphi ={q}_{{\rm{\max }}}\,\cos (x)/m$ which has already been used in the cosmological context to simulate the dynamics of (i) a non-quantum cosmological fluid?, (ii) a Bose-Einstein condensates of axions? Figure 1 shows the evolution of the initial condition (10) through the DTQW for various values of *m* and constant *q*_max_ (the larger the mass, the less relativistic the propagation) and displays multiple shocks. The evolution of (11) is shown in Fig. 2 (compare Fig. 2a,b), which displays a single symmetric shock. Thus, both figures reveal that the DTQW can indeed be used to simulate hydrodynamical shocks in a quantum fluid^29,30^.

Let us stress that the shock is present, not only at high resolutions, but also for resolutions as low as $n=64$ (see Fig. 2), which is well within current experimental limits^31,32^.

### Analytical shock computation in the Galilean regime

We now present an analytical computation which reproduces the shock solution in the Galilean limit where the DTQW becomes a continuous time quantum walk and the Dirac equation goes, as shown in the Supplemental Material, into the ($\hslash =0$) Schrödinger equation $i{\partial }_{t}\psi =-\,\frac{1}{2m}{\partial }_{xx}\psi$.

The Green function for the Schrödinger equation reads

11 $${G}_{0}(x,t|{x}_{0},{t}_{0})=\sqrt{\frac{m}{2i\pi (t-{t}_{0})}}{e}^{\frac{im{(x-{x}_{0})}^{2}}{2(t-{t}_{0})}}.$$

The single-shock solution (${t}_{0}=0$ and ${u}_{{\rm{\max }}}=1$) thus reads

12 $$\psi ({\bf{x}},t)={\int }_{-\infty }^{\infty }\,dy\,\sqrt{\frac{m}{2i\pi t}}{e}^{im(\frac{{(y-x)}^{2}}{2t}+\cos (y))}.$$

In the large-*m* limit, this integral can be computed by making use of methods that are standard in optics^33^ and involve Pearcey’s integral^34^ defined by

13 $${I}_{{\mathscr{P}}}(T,X)={\int }_{-\infty }^{\infty }\,dy{e}^{i(Xy+T{y}^{2}+{y}^{4})}.$$

To wit, we set in the large-*m* limit, $$\psi ({\bf{x}},t)\approx A({\bf{x}},t){I}_{{\mathscr{P}}}(\,-\,T(t),X({\bf{x}},t))$$ with $$T(t)={a}^{-\frac{1}{2}}(\frac{t-1}{2\varepsilon t})$$, $$X({\bf{x}},t)=$$$$-\,{a}^{-\frac{1}{4}}(\frac{x}{\varepsilon t})$$ and $$A({\bf{x}},t)={e}^{i(1+\frac{{x}^{2}}{2t})/\varepsilon }{(2i\pi t\varepsilon \sqrt{a})}^{-1/2}$$ where $$a=m/4!$$ and $$\varepsilon =1/m$$. In this way, Pearcey’s integral Eq. (13) alone can correctly reproduces the structure of the shock (see Fig. 2c).

Useful asymptotic expansions of ${I}_{{\mathscr{P}}}$ are given in^35,36^ and §36.2 of ^37^. In particular, the steepest descent method can be directly used in zone *I* (see Fig. 2d) where $m\gg t/{x}^{2}$. It yields the the following asymptotic form:

14 $${\psi }_{I}({\bf{x}},t)\approx A({\bf{x}},t)\sqrt{\frac{-2i\pi }{{\rm{\Phi }}^{\prime\prime} ({u}_{c})}}{e}^{i{\rm{\Phi }}({u}_{c})}$$

where ${\rm{\Phi }}(u)={u}^{4}-T{u}^{2}+Xu$ and the single saddle-point *u*_*c*_ obeys ${\rm{\Phi }}^{\prime} ({u}_{c})=0$. Near the caustic, in zone *II* of Fig. 2d, 2 new saddle-points appear and the wavefunction can be written in terms of the Airy function $Ai(x)=\frac{1}{\pi }\,{\int }_{-\infty }^{\infty }\,{\rm{dt}}\,\cos (\frac{{t}^{3}}{3}+xt)$. Well inside the caustic in zone *III*, the function can be written as the sum of 3 interfering contributions (see Fig. 2c,d).

**Figure 3:**
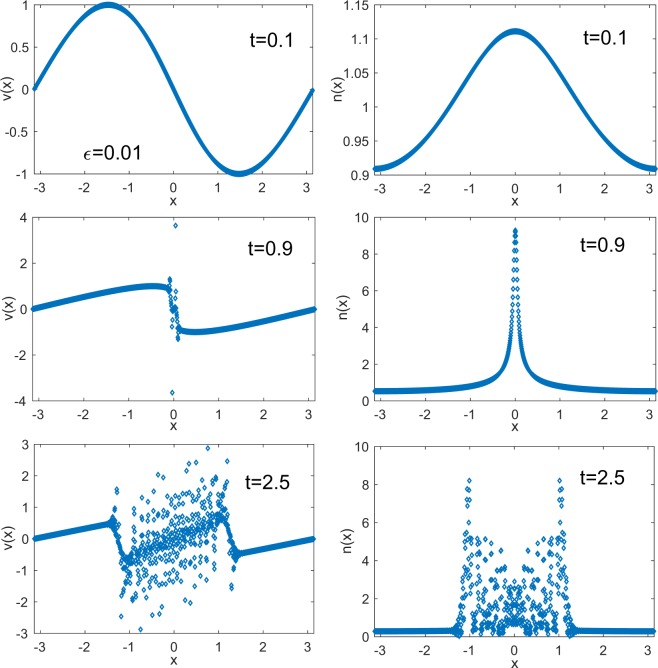
Evolution of velocity $v={m}^{-1}{\partial }_{x}\phi$ (left) and density *n* (right) for 3 values of time and ${m}^{-1}=0.01$ obtained from a numerical solution of Schrödinger equation $i{\partial }_{t}\psi =-\,\frac{1}{2m}{\partial }_{xx}\psi$, with $\psi ={n}^{1/2}\,\exp \,i\phi$ and initial conditions $\phi =m\,\cos \,x$ and $n=1$.

Details of the evolution of the density $n=|\psi {|}^{2}$ and velocity $v={\partial }_{x}\varphi /m$ of the Schrödinger shock are presented in Fig. 3.

## Conclusion

We have shown through a novel generalization of the Madelung transform that one of the simplest DTQWs on the line can be considered as a minimalist model of quantum fluids. This conclusion has been supported by numerical simulations which show that, even within the coherence limit of current experiments, the DTQW evolves an initial condition already considered in the literature into a quantum hydrodynamic shock^29,30^. Thus, under current experimental conditions, DTQW can exhibit hydrodynamical behaviour and, therefore, be used to simulate quantum fluid dynamics. We have also computed the asymptotic shock structure analytically in the non-relativistic limit and proposed an extensive discussion of this limit in the SM.

Quantum walks have already been linked to with hydrodynamics in^38,39^, but these earlier results address the quantum Boltzmann equation and transport phenomena, and are thus quite different from those presented in this Letter.

Note that the quantum fluid described in this article exhibits a non-vanishing quantum pressure but its traditional pressure vanishes identically because the underlying QW is linear. The results presented in this article can be generalised to quantum fluids with non vanishing traditional pressure by working with non-linear QWs such as those already considered in^40–42^. The non-linearity of these walks can be reconciled with the linearity of Quantum Physics by viewing the non-linear terms as an effective description of (self-)interaction, generated for example by the coupling of QWs through gauge fields^21,43–45^. Let us note that non-linear QWs can in principle be realized experimentally, at least through non-linear optics experiments^46,47^.

The present work should be extended to higher dimensions and higher spins. Also, classical pressure terms should be added by considering non-linear DTQWs^41^, or DTQWs with site to site interactions. One should finally incorporate in the Madelung transform the natural coupling of DTQWs to gauge fields^21,44,45,48^, thus obtaining novel models of superconducting quantum fluids or of quantum fluids in relativistic gravitational fields.

## Supplementary information

Supplementary Material
