# Supplementary material for: Quantum walk hydrodynamics
Source: Sci Rep. 2019 Feb 27;9:2989. doi: 10.1038/s41598-019-40059-x (PMC6393481; doi:10.1038/s41598-019-40059-x)
Supplement: Supplementary file 1 — Supplementary Material [file 41598_2019_40059_MOESM1_ESM.pdf]

# Supplementary material to "Quantum walk hydrodynamics"


Mohamed Hatifi
*Aix Marseille Université, Institut Fresnel CNRS UMR 7249, 13013 Marseille, France*

Giuseppe Di Molfetta
*Aix-Marseille Université, Université de Toulon, CNRS, LIS, Marseille, France Natural computation research group and Departamento de Física Teórica and IFIC, Universidad de Valencia-CSIC, Dr. Moliner 50, 46100-Burjassot, Spain*

Fabrice Debbasch
*LERMA, UMR 8112, UPMC and Observatoire de Paris, 61 Avenue de l'Observatoire 75014 Paris, France*

Marc Brachet
*Laboratoire de Physique Statistique, École Normale Supérieure, PSL Research University; UPMC Univ Paris 06, Sorbonne Universités; Université Paris Diderot, Sorbonne Paris-Cité; CNRS; 24 Rue Lhomond, 75005 Paris, France*



This SM provides supplementary information on "Non relativistic limit" of the Quantum Walks Hydrodynamics.


## NON RELATIVISTIC LIMIT

### Wave equation

Dirac equation $i\gamma^\mu \partial_\mu \psi - m\psi = 0$ reads, in component terms and in units where $\hbar = 1$ but $c \neq 1$:

$$\frac{1}{c}\partial_t \Psi_L - \partial_x \Psi_L = -imc\Psi_R$$
$$\frac{1}{c}\partial_t \Psi_R + \partial_x \Psi_R = -imc\Psi_L \qquad (1)$$

Each component obeys the same Klein-Gordon (KG) equation

$$\frac{1}{c^2}\partial_{tt}\Psi_{L/R} - \partial_{xx}\Psi_{L/R} = -m^2c^2\Psi_{L/R}. \qquad (2)$$

The non relativistic limit is obtained as follows. We write $\Psi_{L/R} = \bar{\Psi}_{L/R}\exp(-imc^2 t)$. From now on, all scalings below characterize the action of differential operators on $\bar{\Psi}_{L/R}$. We suppose

$$\frac{1}{mc^2}\partial_t = \mathcal{O}(\nu^2) \qquad (3)$$

where $\nu$ is an infinitesimal. This scaling traces that the energy of $\Psi$ is close to the rest mass energy. The difference will be the kinetic energy which, for a free particle, scales as the squared momentum $p^2$. Thus, $p$ must scale as $\nu$.

Injecting he above scaling in the KG equation for $\bar{\Psi}_{L/R}$ shows that $\bar{\Psi}_{L/R}$ obey the Schrödinger equation when $\nu$ goes to zero. We now also compute for future use the lowest order terms in the difference $\bar{\Psi}_L - \bar{\Psi}_R$. The Dirac equation can be rewritten as

$$\bar{\Psi}_R = \bar{\Psi}_L + \frac{1}{imc}\partial_x\bar{\Psi}_L + \frac{i}{mc^2}\partial_t\bar{\Psi}_L$$
$$\bar{\Psi}_L = \bar{\Psi}_R - \frac{1}{imc}\partial_x\bar{\Psi}_R + \frac{i}{mc^2}\partial_t\bar{\Psi}_R. \qquad (4)$$

In the right-hand sides of the previous equations, the second terms are of order $\nu$ while the third terms are of order $\nu^2$. This is so because $-i\hbar\partial_x$ is the impulse operator and $p = mc + \mathcal{O}(\nu)$ (see remark above). Using the Schrödinger equation makes the orders of the terms more explicit:

$$\bar{\Psi}_R = \bar{\Psi}_L + \frac{1}{imc}\partial_x\bar{\Psi}_L - \frac{1}{2m^2c^2}\partial_{xx}\bar{\Psi}_L + \mathcal{O}(\nu^3)$$
$$\bar{\Psi}_L = \bar{\Psi}_R - \frac{1}{imc}\partial_x\bar{\Psi}_R - \frac{1}{2m^2c^2}\partial_{xx}\bar{\Psi}_R + \mathcal{O}(\nu^3). \qquad (5)$$

### Lagrangian density

Let us start the discussion by keeping only the terms of order $\nu$ in (5). The two wave-function components are equal at order 0 in $\nu$ and thus, at this order, have the same moduli and phases. We want to compute the differences between the moduli and the differences between the phases at first order in $\nu$. This is best done in the following way. Write $\bar{\Psi}_L = r\exp(i\phi)$ and $\bar{\Psi}_R = (r + \delta r)\exp(i(\phi + \delta\phi))$. Thus (see eq. (**??**) and definitions just above) $r^2 = \rho_L$, $(r + \delta r)^2 = \rho_R$, $\phi = \varphi_L - mc^2 t$ and $\delta\phi = \varphi_-$.

Inserting this into (5) and keeping only first order terms leads to:

$$r\exp(i\phi)\left(i\delta\phi + \frac{\delta r}{r}\right) = \frac{1}{imc}\partial_x\bar{\Psi}_L, \qquad (6)$$

from which one gets:

$$\delta\phi = -\frac{1}{2mcr^2}\left(\bar{\Psi}_L^*\partial_x\bar{\Psi}_L + \partial_x\bar{\Psi}_L^*\bar{\Psi}_L\right). \qquad (7)$$

The difference $\delta r$ can be obtained in the same manner:

$$\frac{\delta r}{r} = -\frac{1}{2imcr^2}\left(\bar{\Psi}_L^*\partial_x\bar{\Psi}_L - \partial_x\bar{\Psi}_L^*\bar{\Psi}_L\right). \qquad (8)$$

This transcribes into:

$$\delta\phi = -\frac{1}{mc}\frac{1}{r}\frac{\partial r}{\partial x} \qquad (9)$$

and

$$\frac{\delta r}{r} = \frac{1}{mc}\frac{\partial\phi}{\partial x}. \qquad (10)$$

It is straightforward (but tedious) to compute in the same manner the differences in moduli and phases at second order in $\nu$. One finds:

$$\delta\phi = -\frac{1}{mc}\frac{1}{r}\frac{\partial r}{\partial x} - \frac{1}{2m^2c^2}\frac{\partial^2\phi}{\partial x^2} \qquad (11)$$

and

$$\frac{\delta r}{r} = \frac{1}{mc}\frac{\partial\phi}{\partial x} + \frac{1}{2mc^2}\left(\frac{\partial\phi}{\partial x}\right)^2 + \frac{1}{2m^2c^2r^2}\left(\left(\frac{\partial r}{\partial x}\right)^2 - r\frac{\partial^2 r}{\partial x^2}\right) \qquad (12)$$

Inserting this into the Dirac Lagrangian in hydrodynamical form $\mathcal{L}$ and making use of the equations of motion (4)-(5) of the main text delivers the expression of the Lagrangian density at second order in $\nu$: $\mathcal{L}_{\mathcal{S}} = \mathcal{L}/4 = -(r^2(\partial_x\phi)^2 + (\partial_x r)^2)/2m - r^2\partial_t\phi$ that, using Madelung's relation $\psi = r\exp(i\phi)$, coincides with the usual ($\hbar = 1$) Schrödinger Lagrangian: $\mathcal{L}_{\mathcal{S}} = i(\overline{\psi}\partial_t\psi - \psi\partial_t\overline{\psi})/2 - |\partial_x\psi|^2/2m$. Note that the equations of motion need to be used to pass from the Dirac Lagrangian to the Schrödinger Lagrangian because the two spinor components of the Dirac wave function degenerate into a single Schrödinger wave-function.

### Hydrodynamical variables

At second order in $\nu$ the hydrodynamical variables defined in the main text read:

$$n = 2r^2 + \frac{2r^2}{mc}\frac{\partial\phi}{\partial x} + \frac{1}{m^2c^2}\left[r^2\left(\frac{\partial\phi}{\partial x}\right)^2 + \left(\frac{\partial r}{\partial x}\right)^2 - r\frac{\partial^2 r}{\partial x^2}\right] \qquad (13)$$

$$u^0 = 1 + \frac{1}{2m^2c^2}\left(\frac{\partial\phi}{\partial x}\right)^2 \qquad (14)$$

$$u^1 = \frac{1}{mc}\frac{\partial\phi}{\partial x} + \frac{1}{2m^2c^2r^2}\left[\left(\frac{\partial r}{\partial x}\right)^2 - r\frac{\partial^2 r}{\partial x^2}\right] \qquad (15)$$

$$w = 2mc^2r^2 + 2cr^2\frac{\partial\phi}{\partial x} + \frac{r^2(\frac{\partial\phi}{\partial x})^2 - r\frac{\partial^2 r}{\partial x^2}}{m} \qquad (16)$$
